# Association between lipoprotein(a) plasma levels and diabetic nephropathy in Han Chinese patients with type 2 diabetes mellitus

**DOI:** 10.1371/journal.pone.0299240

**Published:** 2024-05-14

**Authors:** Ting Wang

**Affiliations:** Department of Endocrinology, The First Affiliated Hospital of Anhui Medical University, Hefei, Anhui, People’s Republic of China; Catholic University of Brasilia, BRAZIL

## Abstract

The goal of this study was to evaluate the relationship between serum lipoprotein(a) [Lp(a)] levels and diabetic nephropathy (DN) among Han Chinese individuals with type 2 diabetes mellitus (T2DM). This retrospective analysis comprised a consecutive case series of 767 grown-up patients with T2DM (199 among them with DN) hospitalized in the Department of Endocrinology at the The First Affiliated Hospital of Anhui Medical University from February 20220 to February 2021. Clinical data and other laboratory measurements, such as glycated hemoglobin (HbA1c), were extracted from medical records and compared among groups. Clinical characteristics according to Lp(a) quartiles were also studied. Univariate and multivariate regression analysis were used to examine the relationship between serum Lp(a) and DN. Patients with DN had a longer disease duration, higher HbA1c, higher level of Lp(a), and were more likely to have diabetic retinopathy (DR) than those without DN (P < 0.005 for each). With regard to the Lp(a) quartile group, patients with a higher Lp(a) concentration were more likely to have DN and have higher level of HbA1c during the study (P for trend < 0.005 for each). After adjusting for several confounding factors, the development of DN was significantly associated with the serum Lp(a) level (P = 0.026, comparing the 4th vs 1st quartile of Lp(a)) according to multivariate regression analysis. The receiver operating characteristic (ROC) curves for DN development using serum Lp(a) showed that the area under the receiver operating characteristic curves (AUC) was 0.590 (P < 0.001). Findings from this study demonstrated that the DN was independently associated with the serum Lp(a) level in patients with T2DM in this retrospective study.

## Introduction

Diabetic nephropathy (DN), which is characterized by altered glomerular filtration and proteinuria [1], is as one of the most widely recognized microvascular complications of diabetes and remains the leading causes of end-stage renal disease (ESRD) all around the world [2]. Furthermore, DN is related with unfortunate outcomes and serves as a unique predictor of mortality in diabetes patients [3, 4]. Currently, around 7% of Chinese adults are diabetic, and around one-third of diabetic patients with a history of 10 to 20 years develop diabetic nephropathy [5].

DN is far more difficult to cure than general kidney diseases and its prognosis is poor. Although a number of risk factors for diabetic nephropathy have been presented, including age, duration of diabetes, hyperglycemia, hypertension, and dyslipidemia etc, further detection of residual risk factors for diabetic nephropathy is still of clinical significance for the risk stratification and management of the disease [6, 7].

With a structure similar to low-density lipoprotein (LDL), Lp(a) is mostly composed of cholesterol, triglyceride and apolipoprotein B-100, and contains apolipoprotein (a) [Apo(a)], which is not found in LDL. The entire structure is covalently linked by a single-chain disulfide bond between ApoB-100 and Apo(a). And Lp(a) has also been proven by prospective studies and Mendelian randomization data to be an independent risk factor for atherosclerotic cardiovascular disease (ASCVD) [8]. Microvascular diseases share underlying mechanisms with atherosclerosis and macrovascular disease [9].

Thus, it has been proposed as an independent risk factor for diabetic microvascular complications. Higher serum Lp(a) in patients with T2DM is independently related with higher risks of diabetic nephropathy, according to a Meta-Analysis published recently. Hidenori Senba et al found that serum Lp(a) levels were reported to be positively linked with diabetic nephropathy in Japanese individuals with type 2 diabetes mellitus [10]. Sunny S. Singh et al suggested that Lp(a) plasma levels do not have a significant impact in the development of microvascular complications in people from European descent with type 2 diabetes mellitus [11]. As a result, there is no conclusive evidence of a relationship between Lp(a) and type 2 diabetic nephropathy. To help clarify this discrepancy, we analyzed existing data to study the association between serum levels of Lp(a) and diabetic nephropathy among Han Chinese patients with type 2 diabetes mellitus.

## Methods

### Participants

In this retrospective study, we included data from 767 patients with type 2 diabetes mellitus who were hospitalized in the Department of Endocrinology at Anhui Medical University’s First Affiliated Hospital between February 2020 and February 2021 (the authors accessed the data for research purposes on March 1, 2023, and did not have access to any personal information that could identify individual participants during or after data collection.). All of the people whose data were included in this study were Han Chinese. Exclusion criteria included patients with (1) thyroid abnormalities; (2) any febrile or infectious illness, kidney function abnormalities or uremia, severe heart failure, stroke, cancer, autoimmunological disorders; (3) received hormone replacement therapy or other drugs affecting blood Lp(a) concentrations. DN was defined as microalbuminuria (ACR ≥ 2.5 mg/mmol for men or ≥ 3.5 mg/mmol for women) in at least two of three consecutive measurements or when high microalbuminuria or macroalbuminuria was present at one measurement (ACR ≥ 12.5 for men or ≥ 17.5 for women). It should be noted that not all patients had data for Lp(a) levels due to various circumstances such as unperformed tests or unavailable results. However, these patients were not excluded from the study as they had data for other relevant indicators, such as HbA1c and C-peptide. This study was approved by the Committee on Medical Ethics of the First Affiliated Hospital of Anhui Medical University. The need for participant consent was waived by the committee due to the retrospective nature of the study. All procedures were performed in studies involving human participants in accordance with the ethical standards of the institutional and/or national research committee and with the 1964 Helsinki declaration and its later amendments or comparable ethical standards.

### Clinical and biochemical measurements

Information, including demographic characteristics (age and gender), duration of diabetes mellitus, living habit(smoking and drinking history), medical history (hypertension and hyperlipoproteinemia) and use of medications (insulin, lipid-lowering treatment), was all extracted from the medical records. Height and weight had been measured, and body mass index (BMI) had been calculated using weight over height (kg/m2). Blood pressure (BP) had been taken three times consecutively on the right arm using a mercury sphygmomanometer or an electronic sphygmomanometer after at least five minutes of rest in the sitting position. Fasting blood samples had been obtained from all participants subsequent to refraining from eating, drinking and smoking for at least 8 h. All blood and urine specimens had been tested as soon as they were collected. Immunoturbidimetry had been used to detect lipid profiles and creatinine (Cr); oxidative electrode method had been used to measure fasting blood glucose (FBG); and high-pressure liquid chromatography had been used to measure glycosylated hemoglobin (HbA1c). A chemiluminescent immunometric technique had been used to measure C-peptide levels. Evidence of fatty liver had been analyzed with a medical ultrasonic apparatus. Please note that while these procedures are consistently followed, they may not be explicitly recorded in the medical records of each participant. In addition, the diagnosis and grading of diabetic retinopathy (DR) were performed by qualified ophthalmologists. The diagnosis was based on comprehensive eye examinations, including dilated eye exams and retinal imaging. The grading of DR was performed according to the International Clinical Diabetic Retinopathy Disease Severity Scale [12].

### Statistical analysis

SPSS 22.0 was used to perform the analyses. For continuous variables, descriptive statistics were reported as mean±standard deviation if variables were normally distributed (Shapiro-Wilk test for normality was applied, and P > 0.05 indicated a normally distribution), or as median and interquartile range (25%-75%) if they were skewed. For normally distributed data, Student’s t test was adopted. If the data showed a skewed distribution, Mann-Whitney U test was performed to see whether there were any differences. The categorical variables were tested using χ^2^ tests. And P for trend was estimated for some clinical characteristics of patients according to Lp(a) quartiles. Multivariate regression analyses were conducted to evaluate the relationship between serum Lp(a) concentration and DN. For a more extensive investigation of the connection between Lp(a) and DN, we employed multivariate analysis models to estimate adjusted OR and 95% confidence intervals for DN for the different LP(a) quartiles, using the lowest Lp(a) quartile as a reference. ROC analysis was constructed to evaluate the discriminatory performance for DN presence according to the value of the AUC. In addition to the individual assessments of C-peptide, HbA1c, and Lp(a), a multivariate logistic regression analysis was conducted with DN as the dependent variable and these three variables as independent variables, to evaluate the combined predictive capacity of these variables for DN. The performance of this combined model in predicting DN was also evaluated using the AUC, positive predictive value (PPV), and negative predictive value (NPV). P < 0.05 was viewed as statistically significant.

## Results

A total of 504 men and 263 women, aged 56.10±10.96 years, with a median disease duration of 9 years (interquartile range 3–14 years) were included in the study. A total of 25.9% (199/767) of individuals with type 2 diabetes mellitus were identified with DN. **Table 1** displays the participant characteristics stratified by groups (DN (-) and DN (+)). Patients with DN had had a longer disease duration and were more likely to have DR than those without DN (P < 0.05 for each). The DN (+) group showed greater systolic blood pressure (SBP), HbA1c, and triglycerides (TG), while high-density lipoprotein-cholesterol (HDL-C) was smaller (P < 0.05 for each), indicating a greater metabolic syndrome burden. In addition, patients who developed DN had had a higher Lp(a) than those who did not (P < 0.001). However, age, smoking, drinking, diastolic blood pressure (DBP), BMI, FBG, C-peptide, total cholesterol (TC), and low-density lipoprotein-cholesterol (LDL-C) were not different across groups. Furthermore, patients in the DN (+) group were more likely to utilize insulin and lipid lowering drugs, and less inclined to utilize biguanides than those in the DN (-) group (P < 0.05 for each). The overall population in this cohort had a Lp(a) concentration value of 17.05 mg/dL, with a skewed distribution toward lower concentrations. Patients with a higher Lp(a) concentration were more likely to have had DR (30.2% in 1st quartile of Lp(a) level, 35.6% in 2nd quartile, 38.8% in 3rd quartile, 49.2% in 4th quartile, respectively; P for trend = 0.002) and have higher level of HbA1c during the study (8.00 (7.10–8.90) in 1st quartile of Lp(a) level, 8.50 (7.30–10.08) in 2nd quartile, 8.90 (7.43–10.30) in 3rd quartile, and 8.70 (7.30–10.10) in 4th quartile, respectively; P for trend < 0.001), with regard to the quartile group for the Lp(a). There were also differences in diabetes duration, TG, LDL-C,HbA1c and using biguanides (P fore trend < 0.05 for each); however, there were no distinctions in sex, age, smoking, drinking, SBP, DBP, BMI, FBG, TC, HDL-C, C-Peptide, using insulin, and lipid lowering drugs, according to baseline Lp(a) quartiles (**Table 2**).

**Table 1:** Comparison of baseline parameters between the patients with and without DN.

Characteristics	Total	DN (-)	DN (+)	P value
**Number**	767	568	199	
**Lp(a) (mg/dL)**	17.05 (10.60–27.65)	16.25 (10.13–24.88)	20.30 (12.18–34.30)	<0.001*
**Female, n (%)**	263 (34.3)	201 (35.4)	62 (31.2)	0.279
**Age (y)**	56.10 ± 10.96	55.86 ± 11.15	56.85 ± 10.30	0.247
**Smoking, n (%)**	243 (31.7)	175 (30.8)	68 (34.2)	0.380
**Drinking, n (%)**	224 (29.2)	173 (30.5)	51 (25.6)	0.197
**Diabetes duration (y)**	9.00 (3.00–14.00)	8.0 (3.0–12.0)	10.0 (5.0–17.0)	<0.001*
**BMI (kg/m2)**	25.09 ± 3.55	24.95 ± 3.64	25.42 ± 3.30	0.114
**SBP (mmHg)**	133.00 (122.00–146.00)	131.00 (120.00–144.00)	140.00 (128.00–155.00)	<0.001*
**DBP (mmHg)**	81.00 (74.00–89.00)	81.00 (74.00–89.00)	83.00 (74.00–91.00)	0.193
**HbA1c (%)**	8.40 (7.30–9.80)	8.30 (7.30–9.60)	8.90 (7.58–10.20)	0.002*
**DR, n (%)**	292 (38.1)	177 (31.2)	115 (57.8)	<0.001*
**FBG (mmol/L)**	7.84 (6.27–10.15)	7.83 (6.27–9.87)	7.86 (6.24–11.18)	0.313
**TG (mmol/L)**	1.58 (1.10–2.28)	1.49 (1.07–2.25)	1.68 (1.17–2.37)	0.022*
**C-peptide (ng/ml)**	1.00 (0.64–1.40)	0.99 (0.64–1.37)	1.03 (0.58–1.59)	0.328
**TC (mmol/l)**	4.35 (3.71–5.00)	4.29 (3.72–4.96)	4.56 (3.66–5.15)	0.066
**HDL-C (mmol/l)**	1.02 (0.87–1.23)	1.03 (0.89–1.25)	0.99 (0.82–1.19)	0.009*
**LDL-C (mmol/l)**	2.56 (2.01–3.21)	2.54 (2.02–3.15)	2.72 (1.99–3.48)	0.069
**Insulin, n (%)**	597 (77.8)	430 (75.7)	167 (83.9)	0.016*
**Metformin, n (%)**	494 64.4)	390 (68.7)	104 (52.3)	<0.001*
**Lipid lowering drugs, n (%)**	494 (64.4)	351 (61.8)	143 (71.9)	0.011*

* P<0.05 indicates statistical significance.

Abbreviations: BMI, body mass index; SBP, systolic blood pressure; DBP, diastolic blood pressure; HbA1c, glycated hemoglobin; DR, Diabetic retinopathy; FBG, fasting blood glucose; TG, triglyceride; TC, total cholesterol; HDL-C, high-density lipoprotein cholesterol; LDL-C, low-density lipoprotein cholesterol

**Table 2:** Descriptive characteristics according to the Lp(a) level.

Characteristics	Lp(a)				
	1st quartile	2nd quartile	3rd quartile	4th quartile	P for trend
**Number**	185	193	178	185	
**Lp(a) (mg/dL)**	5.60 (0.15–8.90)	14.10 (12.00–15.70)	21.25 (18.30–23.90)	42.90 (33.70–61.75)	<0.001*
**Female, n (%)**	63 (35.7)	66 (34.2)	59 (33.3)	66 (35.7)	0.966
**Age (y)**	55.77±11.16	54.09±11.57	57.19±10.16	57.01±10.56	0.315
**Smoking, n (%)**	58 (31.4)	66 (34.2)	55 (30.9)	55 (29.7)	0.814
**Drinking, n (%)**	60 (32.4)	56 (29.02)	59 (33.15)	44 (23.8)	0.185
**Diabetes duration (y)**	8.00 (4.00–12.00)	9.00 (3.00–15.00)	10.00 (3.00–14.00)	10.00 (3.50–15.50)	0.454
**BMI (kg/m2)**	25.32±3.44	25.28±4.24	24.97±3.12	24.82±3.22	0.734
**SBP (mmHg)**	134.00 (122.50–147.00)	131.00 (120.00–142.00)	133.50 (120.00–146.00)	136.00 (123.00–154.00)	0.051
**DBP (mmHg)**	82.00 (75.00–90.00)	80.00 (75.00–89.00)	80.00 (73.75–88.25)	81.00 (74.00–89.00)	0.740
**HbA1c (%)**	8.00 (7.10–8.90)	8.50 (7.30–10.08)	8.90 (7.43–10.30)	8.70 (7.30–10.10)	0.001*
**DR, n (%)**	56 (30.2)	69 (35.6)	69 (38.8)	91 (49.2)	0.002*
**FBG (mmol/L)**	7.92 (6.39–9.67)	8.12 (6.49–10.70)	7.72 (6.16–10.25)	7.53 (5.94–9.48)	0.194
**TG (mmol/L)**	1.85 (1.21–2.71)	1.61 (1.13–2.24)	1.40 (1.10–2.14)	1.40 (0.98–2.05)	0.001*
**C-peptide (ng/ml)**	1.07 (0.74–1.48)	0.99 (0.66–1.41)	1.02 (0.65–1.43)	0.85 (0.54–1.35)	0.110
**TC (mmol/l)**	4.24 (3.54–4.88)	4.30 (3.68–4.90)	4.28 (3.73–5.08)	4.55 (3.89–5.16)	0.065
**HDL-C (mmol/l)**	0.98 (0.82–1.16)	1.00 (0.85–1.24)	1.05 (0.91–1.24)	1.04 (0.90–1.25)	0.057
**LDL-C (mmol/l)**	2.36 (1.81–2.88)	2.50 (1.96–3.16)	2.63 (2.07–3.30)	2.84 (2.24–3.38)	<0.001*
**Insulin, n (%)**	139 (75.14)	150 (77.72)	134 (75.28)	154 (83.24)	0.204
**Metformin, n (%)**	132 (71.35)	136 (70.47)	99 (55.62)	110(59.46)	0.002*
**Lipid lowering drugs, n (%)**	109 (58.92)	128 (66.32)	111 (62.36)	128 (69.19)	0.180

* P<0.05 indicates statistical significance.

Abbreviations: BMI, body mass index; SBP, systolic blood pressure; DBP, diastolic blood pressure; HbA1c, glycated hemoglobin; DR, Diabetic retinopathy; FBG, fasting blood glucose; TG, triglyceride, TC, total cholesterol; HDL-C, high-density lipoprotein cholesterol; LDL-C, low-density lipoprotein cholesterol

Age, diabetes duration, FBG, mean HbA1c, DR, DBP, TG, TC, HDL-C and high levels of Lp(a) were all found to be potential risk factors for DN development in a univariate logistic regression analysis. Our analysis also revealed that Drinking was significantly associated with a decreased risk of DN development. (This could be due to potential protective effects of moderate alcohol consumption. However, the detailed exploration of this association and its underlying mechanisms was not within the scope of our current study.) The 2nd, 3rd, and 4th quartiles of Lp(a) were significantly associated with the development of DN during the observation period as compared to the 1st quartile group after adjusting for multiple confounding factors. And the HbA1c remained strong predictive factors for the progression of DN after adjusting for multiple confounding factors (mean HbA1c > 9.0% was substantially linked to the development of DN during the observation period compared to the mean HbA1c < 7.0% group). Furthermore, in multivariable analysis, longer diabetes duration, drinking, higher DBP and more C-Peptide during the research were significant predictors for the development of DN (**Table 3**).

**Table 3:** Multivariable logistic regression model.

	Development of Nephropathy	
	OR (95% CI)	P value
**Lp(a) (mg/dL)**		<0.001$*
**1st quartile**	Reference	
**2nd quartile**	0.482 (0.284–0.818)	0.007*
**3rd quartile**	0.542 (0.328–0.893)	0.016*
**4rd quartile**	0.568 (0.345–0.934)	0.026*
**HbA1c (%)**		<0.001$*
**< 7.0**	Reference	
**7.0–9.0**	0.613 (0.340–1.103)	0.102
**> 9.0**	0.580 (0.373–0.901)	0.015*
**Female, n (%)**	1.510 (0.939–2.428)	0.089
**Age (y)**	0.991 (0.971–1.011)	0.384
**Smoking, n (%)**	1.478 (0.891–2.449)	0.130
**Drinking, n (%)**	0.504 (0.298–0.851)	0.010*
**Diabetes duration (y)**	1.079 (1.079–1.111)	<0.001*
**BMI (kg/m2)**	1.022 (0.968–1.078)	0.438
**SBP (mmHg)**	0.986 (0.966–1.008)	0.207
**DBP (mmHg)**	1.026 (1.013–1.039)	<0.001*
**FBG (mmol/L)**	1.019 (0.951–1.093)	0.590
**TG (mmol/L)**	1.278 (0.992–1.645)	0.057
**C-peptide (ng/ml)**	1.319 (1.044–1.666)	0.020*
**TC (mmol/l)**	0.744 (0.417–1.328)	0.317
**HDL-C (mmol/l)**	0.726 (0.285–1.852)	0.503
**LDL-C (mmol/l)**	1.446 (0.810–2.581)	0.213

* P<0.05 indicates statistical significance.

^$^ P for trend.

Abbreviations: BMI, body mass index; SBP, systolic blood pressure; DBP, diastolic blood pressure; HbA1c, glycated hemoglobin; FBG, fasting blood glucose; TG, triglyceride; TC, total cholesterol; HDL-C, high-density lipoprotein cholesterol; LDL-C, low-density lipoprotein cholesterol

**Fig 1:**
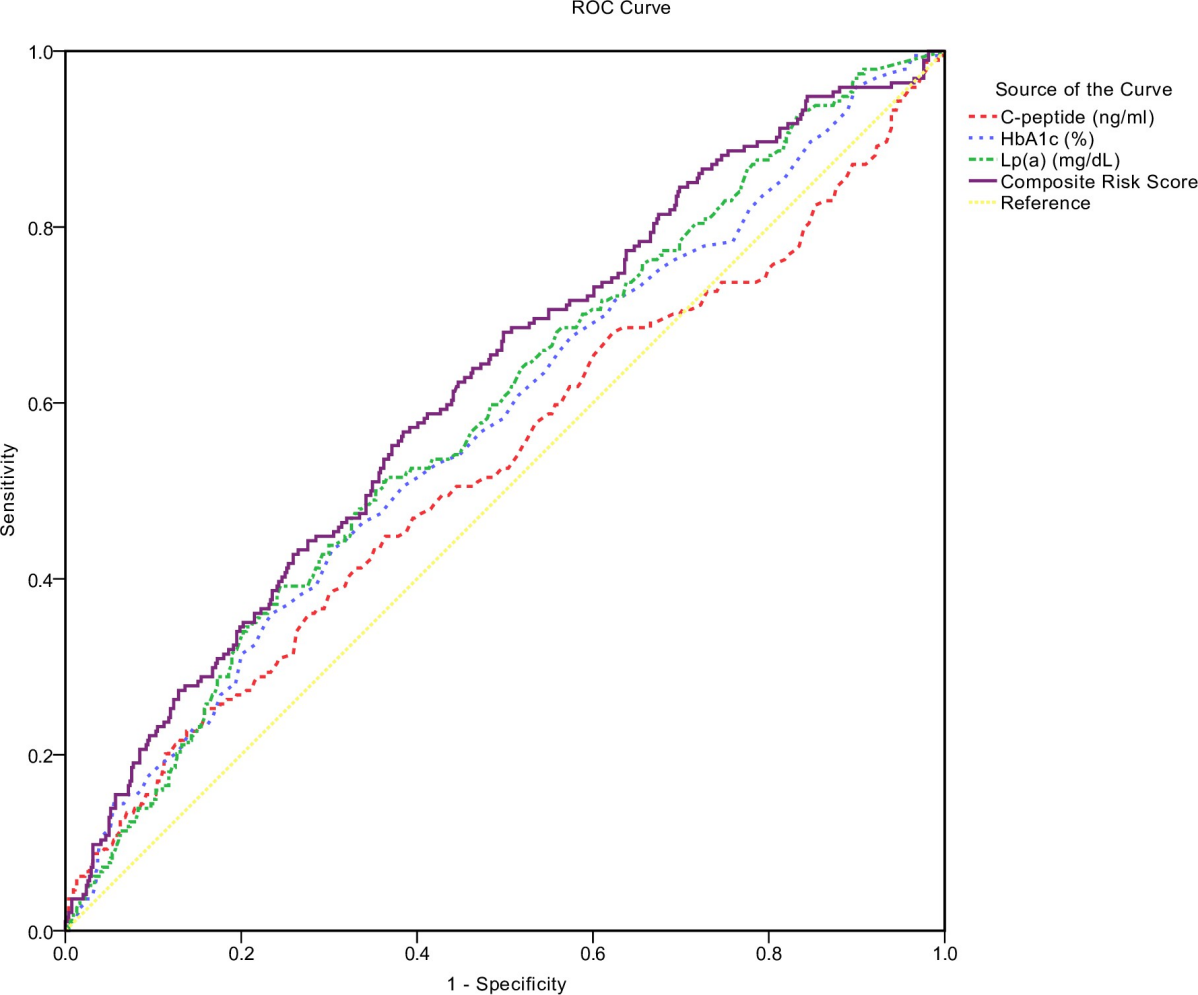
ROC curves for C-peptide, HbA1c, Lp(a), and Composite Risk Score. Receiver operating characteristic (ROC) curves for C-peptide, HbA1c, Lp(a), and the Composite Risk Score to predict the presence of DN in type 2 diabetic patients.

The ROC curves for developing DN revealed that the area under the receiver operating characteristic curves (AUC) using Lp(a) (mg/dL) was significantly higher at 0.590 (P < 0.001), compared to HbA1c at 0.576 (P = 0.002) and C-peptide at 0.532 (P = 0.188) (**Table 4 and Fig 1**). To further investigate the predictive capacity of C-peptide, HbA1c, and Lp(a) for DN, we conducted a multivariate logistic regression analysis with DN as the dependent variable and these three variables as independent variables. The predicted probabilities from this model, which we refer to as “Composite Risk Score”, represent the combined assessment of these variables. When these variables were assessed together in this way, the AUC further increased to 0.619 (P < 0.001) (**Table 4 and Fig 1**). When the Youden Index reached the maximum, the optimal cut-off points for C-peptide, HbA1c, Lp(a), and the Composite Risk Score were defined as > 1.585, > 9.250, > 19.750, and > 0.256, respectively. The corresponding sensitivities and specificities for DN development were 0.253 and 0.892 for C-peptide, 0.433 and 0.697 for HbA1c, 0.515 and 0.634 for Lp(a), and 0.567 and 0.616 for the Composite Risk Score, respectively (**Table 4**). Additionally, the positive and negative predictive values (PPV and NPV) for C-peptide were 0.450 and 0.773, for HbA1c were 0.334 and 0.778, for Lp(a) were 0.330 and 0.789, and for the Composite Risk Score were 0.341 and 0.802, respectively (**Table 4**).

**Table 4:** AUC, cut-off value for C-peptide, HbA1c, Lp(a), and Composite Risk Score.

	Cut-off Value	Sensitivity	Specificity	Youden Index	PPV	NPV	AUC	95% CI	P value
**C-peptide (ng/ml)**	1.585	0.253	0.892	0.095	0.450	0.773	0.532	0.482–0.582	0.188
**HbA1c (%)**	9.250	0.433	0.697	0.130	0.334	0.778	0.576	0.529–0.624	0.002*
**Lp(a) (mg/dL)**	19.750	0.515	0.634	0.150	0.330	0.789	0.590	0.543–0.638	<0.001*
**Composite Risk Score** ^**$**^	0.256	0.567	0.616	0.183	0.341	0.802	0.619	0.573–0.665	<0.001*

AUC, cut-off value for C-peptide, HbA1c, Lp(a), and Composite Risk Score to predict the presence of DN in patients with T2DM.

Composite Risk Score^$^: The Composite Risk Score represents the predicted probabilities from a multivariate logistic regression model with DN as the dependent variable and C-peptide, HbA1c, and Lp(a) as independent variables.

* P<0.05 indicates statistical significance.

Abbreviations: PPV, positive predictive value; NPV, negative predictive value

## Discussion

In this retrospective study, we assessed the relationship between the serum Lp(a) level and the presence of DN. Patients with DN were found to have higher Lp(a) levels than those without the disease. This observation aligns with the hypothesis that elevated Lp(a) levels may contribute to the pathogenesis of DN, possibly through promoting atherosclerosis or inducing renal inflammation, although the exact mechanisms remain to be elucidated. Multivariate analysis revealed that serum Lp(a) was independently associated with the presence of DN in patients with T2DM, suggesting that Lp(a) could be a risk factor for DN beyond the traditional risk factors such as hyperglycemia and hypertension. However, the causal relationship between Lp(a) and DN needs to be confirmed by prospective studies. And, patients with the highest quartile of Lp(a) levels had had a greater risk of DN, according to this study, indicating a dose-response relationship between Lp(a) levels and DN risk. This relationship could have important implications for risk stratification and management of patients with T2DM. Our study demonstrated that Lp(a) had a superior performance in predicting DN, with a higher ROC AUC score (AUC 0.590, P < 0.001) compared to HbA1c (AUC 0.576, P = 0.002) and C-peptide (AUC 0.532, P = 0.188) in patients with type 2 diabetes mellitus. This suggests that Lp(a) could be a more reliable biomarker for DN, although the clinical significance of this difference needs further investigation. It would be interesting to explore whether incorporating Lp(a) into existing risk prediction models could improve their performance. The optimal cut-off point for Lp(a) was defined as > 19.750, suggesting its potential as a valuable indicator for DN. This cut-off point was determined based on our data, but its applicability in different populations and settings needs to be validated. It’s also worth investigating whether different cut-off points should be used for different subgroups, such as patients with different ages or comorbidities. Despite its lower AUC, C-peptide, along with HbA1c, still showed some predictive value for DN. The positive and negative predictive values (PPV and NPV) for C-peptide were 0.450 and 0.773, for HbA1c were 0.334 and 0.778, and notably, for Lp(a) were 0.330 and 0.789, respectively. When these variables were assessed together in a multivariate logistic regression model, the AUC further increased to 0.619 (P < 0.001), indicating an improved predictive capacity for DN. The PPV and NPV for this combined model were 0.341 and 0.802, respectively. Despite the lower PPV for Lp(a), indicating a limited ability to confirm DN in patients with positive test results, its higher NPV suggests a strong ability to rule out DN in patients with negative test results. These values should be interpreted in the context of the prevalence of DN in the tested population. These findings underscore the potential of Lp(a), along with HbA1c and C-peptide, as valuable indicators for DN, with the combined assessment showing the most promise. However, the optimal combination and weighting of these markers need to be determined in future studies.

Previous findings indeed support our conclusion that Lp(a) levels are associated with DN [13, 14, 16–19]. For instance, Yun et al. found a link between Lp(a) plasma levels and diabetic nephropathy in a South Korean population in their prospective studies [13]. Moosaie F et al discovered that serum Lp(a) level was a predictor of nephropathy in 1057 Iranian patients with type 2 diabetes mellitus in a case-cohort study [14]. In diabetic individuals, elevated Lp(a) levels may be associated with renal failure or increased albuminuria [15, 16]. Lp(a) was found to be a significant prognostic factor for the development of a new onset of CKD in diabetic patients in several studies [17, 18]. However, it’s crucial to acknowledge that some studies have shown conflicting results [12, 19]. For instance, a study found no significant association between baseline Lp(a) levels and renal function decline, cardiovascular events, or mortality in individuals with type 2 diabetes and microalbuminuria [19]. This inconsistency in study outcomes could be attributed to a variety of factors, including ethnic variances in plasma levels of Lp(a), differences in study design, sample selection, and data analysis methods. The role of genetic and environmental variables in explaining these ethnic variances is still a subject of ongoing debate [20]. Indeed, while genetic factors such as the size polymorphism of the LPA gene significantly influence Lp(a) levels, environmental conditions, age, sex, hormonal impact, and certain clinical conditions like kidney and liver diseases also contribute to the observed variability in Lp(a) levels among different populations [20]. Lp(a) is highly heritable, which is determined by race-determined differences and copy-number variation at the LPA locus on chromosome [21]. Lp(a) levels are inversely correlated with the size of the genetically determined Apo(a) subtype, higher plasma Lp(a) levels are related with smaller Apo(a) subtype size, and there may be significant variability in Lp(a) levels amongst people with a given Apo(a) subtype size [22]. However, the exact mechanisms and implications of these genetic variations are not fully understood and warrant further investigation. The association between lower eGFR and higher serum Lp(a) levels, particularly in non-Hispanic blacks, suggests ethnic differences, but the underlying causes and implications of these differences need to be further explored [23]. Furthermore, while several studies have found that blacks had higher levels of Lp(a) than whites, and significant heterogeneity in Asian groups has been documented, with Chinese populations having lower levels of Lp(a) than Indian populations [24, 25], these findings are not universally accepted. Some studies have reported conflicting results, and the reasons for these discrepancies are not well understood [26, 27]. Indeed, it has been established that race/ethnic differences in plasma Lp(a) concentration and population distribution exist, with Blacks having the highest Lp(a) level of all race/ethnic groups studied [26]. However, the lack of standardization in the commercial assays used to measure Lp(a) levels makes it difficult to assess risk based on individual Lp(a) levels, highlighting the need for more studies in diverse populations [27].

In our retrospective analysis, we found that plasma Lp(a) levels reflect a balance of Lp(a) synthesis, which happens in the liver, and catabolism, which is assumed to involve the kidney [28]. This balance can be influenced by various factors, including renal function and protein loss. A study involving 224 patients diagnosed with diabetic kidney disease (DKD) found significant differences in Lp(a) levels among different chronic kidney disease (CKD) stages, and Lp(a) levels correlated with several renal function-related indicators [29]. However, it’s important to note that the exact mechanisms underlying these associations are not fully understood and warrant further investigation. On one hand, the kidney is thought to be a site of Lp(a) catabolism, and pathological alterations in the kidney, such as those occurring in end-stage renal disease, might lead to decreased excretion of Lp(a) and thus increased Lp(a) levels [30]. A study involving a large sample of Chinese adults found that Lp(a) levels were independently associated with several renal function indicators, including estimated glomerular filtration rate (eGFR), serum creatinine (Scr), and blood urea nitrogen (BUN) [31]. However, the relationship between renal function and Lp(a) levels is a subject of ongoing debate, with some studies reporting conflicting results [32]. These discrepancies could be due to differences in study design, sample selection, or data analysis methods, and highlight the need for further research in this area. On the other hand, conditions that lead to significant protein loss, such as nephrotic syndrome or peritoneal dialysis treatment, might trigger the liver to ramp up protein synthesis, including that of Lp(a) [33, 34]. This notion is backed by the research conducted by Hopewell et al., who discovered a correlation between increased Lp(a) levels and nephropathy, but only in patients with larger Apo(a) subtypes [28]. The clinical implications of this association, particularly how it might influence the management of patients with DN, remain unclear and necessitate further exploration [35]. These findings underscore the intricate nature of the relationship between Lp(a) levels and DN, emphasizing the need for comprehensive studies that take into account a broader spectrum of factors.

Our retrospective study provides evidence that elevated Lp(a) levels are associated with the presence of diabetic nephropathy (DN).Patients with DN in our study had higher Lp(a) levels than those without the disease. This observation aligns with the known biological mechanisms of Lp(a). Lp(a) is known to have an atherogenic effect and could play a role in the evolution of diabetic nephropathy via atherosclerosis [36]. For instance, Lp(a) might promote vascular stenosis, trigger hypertension leading to renal damage, and induce endothelial cell apoptosis through oxidative stress [37–39]. The accumulation of Lp(a) in the arterial intima is a characteristic of atherosclerosis, and its atherogenicity could be enhanced by covalent linkage with apo(a) or through oxidation [37]. Furthermore, both Lp(a) and oxidized Lp(a) can induce the production of reactive oxygen metabolites in glomeruli, potentially through a pathway that is sensitive to the inhibition of protein kinase C and the elevation of intracellular cAMP levels [38]. These reactive oxygen metabolites could further induce endothelial cell apoptosis, thereby influencing the pathogenesis of atherosclerosis [39]. Furthermore, Lp(a) is associated with inflammatory conditions [40], and its component, Apo(a), is a proinflammatory molecule that directly interacts with the leukocyte β2-integrin MAC-1, thereby encouraging the accumulation of inflammatory cells [41]. This could potentially explain the higher incidence of DN in patients with elevated Lp(a) levels in our study. Moreover, Lp(a) is linked to inflammatory conditions, acting through its component, Apo(a), to interact with the leukocyte β2-integrin MAC-1, promoting monocyte adhesion and migration [40]. This interaction, intensified by proatherogenic homocysteine, could partly explain the higher DN incidence in patients with elevated Lp(a) levels in our study. Additionally, chronic inflammation, often seen with high Lp(a) levels, contributes to residual cardiovascular risk [41]. However, the role of inflammation in DN is complex, involving multiple pathways, including those related to Type 2 Diabetes Mellitus [42] and its associated complications, such as chronic and low-grade inflammation [43]. Certain inflammatory markers, such as serum uric acid-based indicators [44], C-reactive protein-based markers [45], and high-sensitivity C-reactive protein [46], have been linked to DN. These markers, including neuregulin-4 [42], the systemic inflammatory index [43], Uric Acid to HDL ratio [44], C-reactive protein to serum albumin ratio [45], and high-sensitivity C-reactive protein [46], have shown significant correlations with DN. However, the causal relationships remain unclear and necessitate further investigation. These findings suggest that inflammation could play a significant role in the development of DN, but the exact mechanisms and pathways involved are still not fully understood. In conclusion, our study provides evidence that elevated Lp(a) levels may contribute to the development of DN, possibly through the mechanisms discussed above. However, further research is needed to confirm these mechanisms and to explore other potential pathways.

In this retrospective study, we investigated the relationship between Lp(a) concentration and DN in Chinese patients. Our results indicate that Lp(a) might serve as a potential biomarker for the detection of diabetic nephropathy in Han Chinese patients with type 2 diabetes. However, several limitations of this study should be acknowledged when interpreting our findings. Firstly, the nature of our study being retrospective observational limits our ability to establish direct causality between Lp(a) levels and DN. Future prospective studies are needed to confirm this relationship and to explore the potential causal mechanisms. Secondly, the wide and biased range of Lp(a) plasma concentrations, coupled with the lack of a standard method for determining Lp(a), may have influenced our results. Future research should aim to standardize the measurement of Lp(a) [47], and to explore the potential impact of measurement bias on the association between Lp(a) and DN. Thirdly, our inability to measure the Apo(a) subtype and Lp(a) genotype at baseline could have potentially influenced our understanding of the relationship between Lp(a) and DN. Future studies should consider including these measurements to provide a more comprehensive understanding of the role of Lp(a) in DN. Fourthly, we did not consider all potential confounding factors in our multivariate analysis, which might have influenced the observed association between Lp(a) and DN. Future studies should consider using more complex statistical models to control for more confounding factors. Lastly, the significant differences in Lp(a) concentrations between ethnic groups suggest that our findings may not be generalizable to all ethnic populations. Our study population consisted of consecutively included hospitalized Han Chinese patients with type 2 diabetes from a specific location. Future studies should consider including a more diverse population to improve the generalizability of the findings. Despite these limitations, we propose that Lp(a) could be a significant risk factor for DN. We recommend that type 2 diabetes patients, especially those in the highest interquartile range of Lp(a), should maintain strict glycemic control and undergo regular renal function monitoring.

## Conclusion

Despite these limitations, we propose that serum concentrations of Lp(a) were associated with DN in Han Chinese patients with T2DM based on our retrospective study. To determine if serum levels of Lp(a) were associated to the beginning or progression of diabetic nephropathy, more evidence from a well-designed prospective trial would be needed.
